# Follow-up for 3 years of a pediatric population diagnosed in 2018 with mother-to-child transmission of HIV in 8 Latin American countries in the PLANTAIDS cohort

**DOI:** 10.1186/s12879-024-09091-9

**Published:** 2024-02-20

**Authors:** Beatriz Álvarez Vallejo, Alicia Hernanz Lobo, Itzíar Carrasco García, Tomás Bruno Pérez, Greta Mino-Leon, Judith Rosabel Soffe Pazmiño, Julio Werner Juarez Lorenzana, Tatiana Drummond, Noris Marlene del Socorro Pavía Ruz, María del Rocío Muñoz Hernández, Dulce María Morales Pérez, Dora Estripeaut, Kathia Luciani, Karen Sobeida Erazo Martínez, Luis Guillermo Castaneda Villatoro, Oscar Porras Madrigal, Gabriela Ivankovich-Escoto, Luis Manuel Prieto Tato, María Luisa Navarro Gómez, on behalf of the PLANTAIDS network, Dora Matus Obregón, Pablo Rojo Conejo, José Tomás Ramos Amador, Magda Chavez, María de los Ángeles Pérez Delgadillo, Fanny Barrios, Osbaldo Efraín Obando Urbina, Greta Muñoz, Wendy Paola Serrano Bueno, Dolores Freire, Nelly Chavez, Yasmin Sanchez, Marianella Layana, Alexandra Compagnucci, Yacine Saïdi, Yoann Riault, Guido Castelli Gattinara, Tchidjou Kuekou Hyppolite, Raúl Esquivel, Ruth Batista, Ximena Norero, Jacqueline Aguilar, Ilia Yanira González, Gustavo Quiñonez, Ana Lucía Gómez, Nancy Judith Gálvez Rafael

**Affiliations:** 1 Pediatric Infectious Diseases Department. Hospital Clínico, Universitario Virgen de La Arrixaca, Murcia, Spain; 2 Pediatric Infectious Diseases Department, Gregorio Marañón University Hospital, Madrid, Spain; 3 Gregorio Marañón Research Health Institute (IiSGM), Madrid, Spain; 4 Centro de Investigación Biomédica en Red de Enfermedades Infecciosas (CIBERINFEC), Instituto de Salud Carlos III, Madrid, Spain; 5 RITIP Translational Research Network in Pediatric Infectious Diseases, Madrid, Spain; 6 Internal Medicine Department, Hospital Universitario Santa Lucía, Cartagena, Spain; 7 Infectious Diseases Service. Hospital del Niño Dr, Francisco de Icaza Bustamante, Guayaquil, Ecuador; 8 Unidad de Atención Integral del VIH e Infecciones Crónicas. Hospital Roosevelt, Guatemala City, Guatemala; 9 Pediatric Infectious Disease Service. Department of Paediatrics, Hospital Universitario de Caracas, Caracas Capital District, Venezuela; 10 Paediatric HIV/AIDS Clinic, UNAM/HGM, Facultad de Medicina, Universidad Nacional Autónoma de México, Mexico City, México; 11 Pediatric Infectious Diseases Department, CLINDI, Hospital Infantil de México Federico Gómez, Mexico City, México; 12 Pediatric Infectious Disease Service, Hospital del Niño Dr. José Renán Esquivel, Ciudad de Panamá, Panamá; 13 Sistema Nacional de Investigación (SNI) de la Secretaría Nacional de Ciencia y Tecnología (SENACYT), Ciudad de Panamá, Panamá; 14 Pediatric Infectious Disease Service, Hospital de Especialidades Pediátricas Omar Torrijos Herrera, Ciudad de Panamá, Panamá; 15 Department of Paediatrics., Hospital Dr Mario Catarino Rivas, San Pedro Sula, Honduras; 16 Pediatric HIV/AIDS Clinic, Hospital Nacional de Niños Benjamín Bloom, San Salvador, El Salvador; 17 Department of Paediatrics, Hospital Nacional de Niños Dr. Carlos Sáenz Herrera, San José, Costa Rica; 18 Department of Paediatrics, Hospital Doce de Octubre, Madrid, Spain; 19 CYTED (Ibero-American Programme of Science and Technology for Development) , https://www.cyted.org/es/plantaids; 20 Universidad Complutense de Madrid (UCM), Madrid, Spain

**Keywords:** HIV/AIDS, Children, Mother‐to‐child transmission, SARS-CoV-2 infection

## Abstract

**Introduction:**

The frequency of mother-to-child transmission (MTCT) of human immunodeficiency virus (HIV) in Latin America has decreased considerably. However, new infections continue to be recorded, and the pediatric population remains one of the most vulnerable groups in this region. The main objective of the study was to describe the clinical, epidemiological and psychosocial characteristics of new diagnoses of HIV MTCT in 2018 in the PLANTAIDS network (Paediatric Network for Prevention, Early Detection and Treatment of HIV in Children) during the 3 years following diagnosis.

**Methodology:**

Retrospective, multicenter, descriptive study based on a 3-year follow-up of patients diagnosed with HIV infection due to MTCT in 2018 in 10 hospitals in 8 Latin American countries (Costa Rica, Ecuador, Mexico, Honduras, El Salvador, Panama, Guatemala and Venezuela). The hospitals belonged to the PLANTAIDS network, which is included in CYTED (Ibero-American Programme of Science and Technology for Development).

**Results:**

The study population comprised 72 pediatric patients (38.9% male). The median age at diagnosis was 2.4 years (IQR: 0.8–5.4). There were 35 cases of opportunistic infections corresponding to 25 patients (34.7%), with tuberculosis being the most common. Adequate childhood vaccination coverage was achieved in 80.5%. There were 3 cases of acute SARS-CoV-2 infection, and these were asymptomatic or mildly symptomatic. According to the *Centers for Disease Control and Prevention* (CDC) classification, the most frequent clinical-immunological stage at all check-ups was C1. Three patients died from opportunistic infections and/or advanced HIV infection.

**Conclusions:**

It is important to diagnose HIV infection early in pediatrics, since early initiation of ART is associated with a decrease in mortality. Despite this, HIV infection has a poor prognosis in children, necessitating adequate follow-up to ensure adherence to health care and ART, although it can sometimes prove difficult in children.

## Introduction

According to data from the Joint United Nations Program on HIV/AIDS (UNAIDS) [1], new HIV infections in children worldwide were reduced by more than half (54%) between 2010 and 2020, mainly owing to increased antiretroviral coverage in pregnant women. However, this trend slowed during the COVID-19 pandemic, with an estimated 400 new infections occurring every day in children under 15 years of age in 2020, leading to a total of 150 000 new infections and 99 000 deaths due to HIV in the pediatric age group.

Consistent with data from throughout the world, the rate of mother-to-child transmission (MTCT) of HIV has declined considerably in recent years (from 20.01% in 2010 to 14.07% in 2018) [2]. Nevertheless, the pediatric population remains one of the most vulnerable groups in the region and faces great difficulties in reaching the 95–95-95 targets set by UNAIDS to end the AIDS epidemic.

During the first 5 years of life, disease progression can be particularly rapid [3]; therefore, early diagnosis and initiation of antiretroviral treatment (ART) are essential [4]. Subsequent adherence to ART will play a key role in the efficacy of the antiviral response.

This study provides follow-up data for a Latin American cohort of children diagnosed with MTCT HIV infection in 2018 [5]. The main objective of the study was to describe the clinical, analytical, and psychosocial characteristics of new diagnoses of MTCT in 2018 in the PLANTAIDS Network of the CYTED program (Ibero-American Programme of Science and Technology for Development) 3 years after diagnosis.

The secondary objective was to evaluate the relationship between early diagnosis (less than 1 year) and the clinical and immunological progress of patients.

## Methods

### Design

Retrospective, multicenter, analytical, and descriptive study on the 3-year follow-up of patients diagnosed with HIV infection due to MTCT in 2018 in 10 hospitals in 8 countries (Costa Rica, Ecuador, Mexico, Honduras, El Salvador, Panama, Guatemala, and Venezuela) belonging to the PLANTAIDS network of the CYTED [5] program.

### Variables

Variables were recorded in a REDCap (Research Electronic Data Capture) database and included information about treatment, clinical and immunological status, opportunistic infections, SARS-CoV-2 infection, and mortality from diagnosis to 3 years of follow-up. To this end, 4 successive check-ups were proposed. The first covered diagnosis to 6 months, the second from 7 to 12 months, the third from 13 to 24 months, and the fourth from 25 to 36 months after diagnosis.

To establish the clinical and immunological stage of the participants, we used the 1994 revised classification system for HIV infection in children less than 13 years of age (CDC).

The World Health Organization (WHO) child growth standards were used to analyze the anthropometry of the patients at each check-up, using the weight and height percentile in children under 5 years of age and the body mass index (BMI) in children over 5 years of age.

### Statistical analysis

Continuous variables were described using the median and interquartile range (IQR); qualitative variables were described using frequencies and percentages. Quantitative variables were compared using the *t* test in the case of parametric variables and the Mann–Whitney test in the case of non-parametric variables. The Wilcoxon test was used for paired data comparisons. Regarding the qualitative variables, the chi-square test was used for parametric variables, Fisher's exact test for non-parametric variables, and McNemar's test for paired data. In all comparisons, an alpha error of less than 0.05 was used to establish statistical significance.

### Definitions

*Adherence to antiretroviral therapy (ART):* “good adherence” was defined as taking the medication on more than 90% of the days, based on the patient's or caregiver's self-reported adherence.

*Psychomotor development:* “normal” psychomotor development was defined as the correct acquisition of developmental milestones according to the patient's age based on the physician’s criteria; any other types of psychomotor development were considered “pathological”.

*Vaccination:* “correct vaccination” was defined as age-appropriate immunization according to the vaccination schedule in each country of origin.

*Early HIV diagnosis:* “Early diagnosis” was defined as that made during the first year of the patient's life.

## Results

**Fig. 1:**
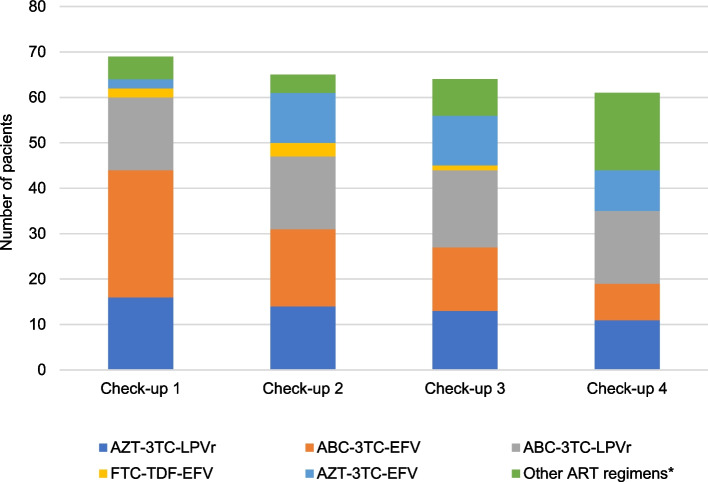
ART regimens in force at each of the check-ups Legend: Other ART regimens*: ABC (abacavir)-3TC (lamivudine)-DTG (dolutegravir), ABC-3TC-RTV (ritonavir), AZT (zidovudine)-ABC-3TC, ABC-3TC-NVP (nevirapine), AZT-3TC-RTV, 3TC-TDF (tenofovir disoproxil fumarate)-EFV (efavirenz), 3TC-TDF-DTG, AZT-ABC-EFV, AZT-FTC (emtricitabine)-DTG, BIC (bictegravir)-TAF (tenofovir alafenamide)-FTC, AZT-3TC-DTG. *Check-up 1: diagnosis - 6 m* *Check-up 2: 7 m - 12 m* *Check-up 3: 13 m - 24 m* *Check-up 4: 25 m - 36 m*

The study population comprised 72 pediatric patients (38.9% male) diagnosed with HIV infection by MTCT during 2018 in the PLANTAIDS Network. Regarding their countries of origin, 21 were from Ecuador, 14 from Mexico, 14 from Guatemala, 7 from Panama, 6 from El Salvador, 5 from Honduras, 3 from Venezuela, and 2 from Costa Rica. The median age at diagnosis was 2.4 years (IQR: 0.8–5.4). Twenty-three patients (31.9%) were diagnosed in the first year of life. According to the CDC classification for people living with HIV [6], the most frequent clinical-immunological stage at all check-ups was C1. ART was prescribed early after diagnosis in all patients, except for one (aged 3.3 years and clinically asymptomatic [stage N1]), in whom it was decided to temporarily postpone initiation owing to the unavailability of a caregiver at the time (mother with cerebral toxoplasmosis, father not present). The ART regimens in force at each check-up, chosen according to the treatment guidelines and strategies of each country, are presented in Fig. 1.

ART-induced toxicity was reported on 15 occasions during follow-up (15/260, 5.8%), lipid profile abnormalities being the most frequent (46.7%), followed by diarrhea, vomiting, anemia, and rash. On 6 of the 7 occasions when lipid profile alterations were identified, the patient was receiving a regimen that included lopinavir boosted with ritonavir (LPV/r), and on 1 occasion efavirenz (EFV). No ART regimen was changed based on lipotoxicity.

A total of 35 opportunistic infections were reported at the 4 check-ups (25 patients), the most frequent being tuberculosis, with 11 cases (31.43%). The patients with suspected tuberculosis were from 3 countries (Panama, Mexico, and Ecuador). The index case was known in 5 cases (5/11, 45.5%). Based on the symptoms, there were 4 cases of suspected disseminated disease (4/11, 36.4%). In all 3 countries, the diagnosis was established using GeneXpert MTB. However, microbiological confirmation was only achieved in 1 patient (positive smear microscopy, culture, and GeneXpert sputum). Neither the interferon gamma release assay (IGRA) nor the lateral flow urine lipoarabinomannan assay (LF-LAM) was performed owing to lack of availability. The remaining opportunistic infections that occurred during follow-up included cytomegalovirus infection (14.3%), *Pneumocystis jirovecii* infection (2.9%), and "other opportunistic infections" (eg, pneumonia, otitis, abscess, varicella, histoplasmosis) in 51.4%.

Regarding active immunization, of the patients who completed follow-up, adequate vaccination coverage was recorded in 80.5% (33/41) at the last check-up, according to age and country of origin. As for vaccination, 92.9% (39/42) were fully vaccinated against hepatitis B virus (HBV), 87.8% (36/41) against diphtheria-tetanus-pertussis (dTP), 92.7% (38/41) against *Haemophilus influenzae* (Hib) and 90.2% (37/41) against pneumococcus. In the case of pneumococcus, all patients received a conjugate vaccine (no polysaccharide vaccines were administered against pneumococcus). In all, 85.4% (35/41) received at least 1 dose against rotavirus, and 31% (13/42) received at least 1 dose against hepatitis A virus. Vaccination against poliomyelitis was by attenuated vaccine (oral polio) in 10.3% of cases (4/39), by inactivated vaccine (parenteral polio) in 41% (16/39), and by administration of both types of vaccine in 48.7% (19/39). Regarding live vaccines, 97.6% (40/41) received 1 or 2 doses of measles-rubella-mumps vaccine (MMR), 24.4% (10/41) were vaccinated against varicella, and 27.9% (12/43) against yellow fever. Vaccination against tuberculosis or bacillus Calmette-Guérin (BCG) was administered in 90% (36/40) of cases. Of the 11 patients in whom tuberculosis was suspected, 10 (90.9%) had received BCG at birth, with no evidence of subsequent lymphadenitis or disseminated disease ("*BCGitis"*).

Regarding feeding mode, 45.5% (30/66) were breastfed, with a median duration of 8 months (IQR: 5–18).

**Fig. 2:**
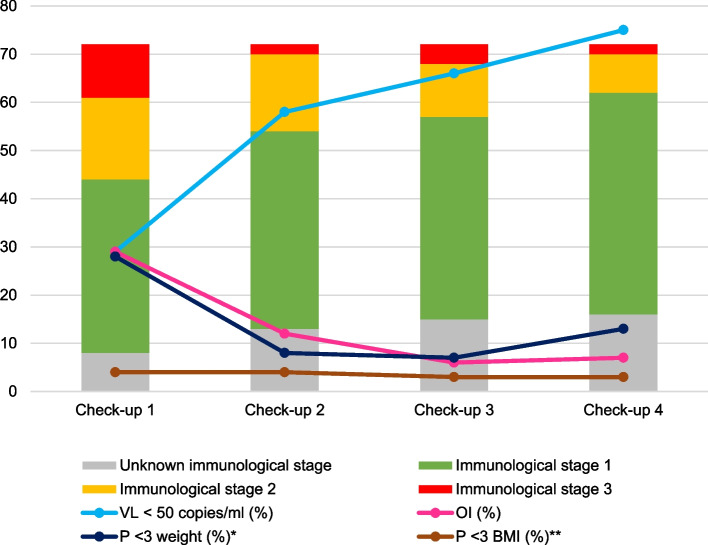
Evolution of the CDC immunological classification, viral load, opportunistic infections, and weight during follow-up *Legend*: *VL: viral load* *OI: opportunistic infections* *P: percentile* * Only in children under 5 years ** Only in children over 5 years * Check-up 1: diagnosis - 6 m* * Check-up 2: 7 m - 12 m* * Check-up 3: 13 m - 24 m* * Check-up 4: 25 m - 36 m*

As for immunologic status, 74.6% of patients had a viral load < 50 copies/ml and immunologic stage 1 at the last check-up. The remaining characteristics of treatment and clinical and immunologic status at each of the check-ups are detailed in Table 1. Likewise, Fig. 2 shows the different immunological stages during follow-up, as well as other parameters (viral load, opportunistic infections, and weight).

**Table 1:** Clinical and immunological characteristics, treatment, SARS-CoV-2 infection, and psychosocial care at each of the check-ups

	**Check-up 1** (diagnosis – 6 m)	**Check-up 2** (7 m – 12 m)	**Check-up 3** (13 m – 24 m)	**Check-up 4** (25 m – 36 m)
**Age (years)** Median (IQR)	3 (1.1–6.05)	3.75 (1.80–8.08)	4.95 (2.73–9.03)	5.95 (4.20–9.08)
**ART**	68/69 (98.55%)	65/65 (100%)	64/64 (100%)	61/61 (100%)
**Good adherence**	57/67 (85.07%)	52/61 (85.25%)	53/64 (82.81%)	55/60 (91.67%)
**CD4 (cells/µl)** Median (IQR)	875 (540–1338)	1084 (763–1492.5)	985 (696–1259)	1102 (821.5–1371.25)
**Viral load < 50 copies/ml**	20/68 (29.41%)	34/59 (57.63%)	40/61 (65.57%)	44/59 (74.57%)
**Opportunistic infections**	28/72 (29.17%)	8/65 (12.31%)	4/61 (6.56%)	4/62 (6.45%)
**SARS-CoV-2 infection**	0/43 (0%)	0/62 (0%)	0/41 (0%)	3/43 (6.98%)
**Clinical stage N**^**a**^	9/71 (12.67%)	7/63 (11.11%)	7/64 (10.94%)	7/58 (12.07%)
**Clinical stage A**^**a**^	16/71 (22.53%)	16/63 (25.40%)	18/64 (28.13%)	17/58 (29.31%)
**Clinical stage B**^**a**^	12/71 (16.90%)	11/63 (17.46%)	9/64 (14.06%)	8/58 (13.79%)
**Clinical stage C**^**a**^	34/71 (47.89%)	29/63 (46.03%)	30/64 (46.88%)	26/58 (44.83%)
**Immunological stage 1**	36/64 (56.25%)	41/59 (69.49%)	42/57 (73.68%)	46/56 (82.14%)
**Immunological stage 2**	17/64 (26.56%)	16/59 (27.12%)	11/57 (19,30%)	8/56 (14.29%)
**Immunological stage 3**	11/64 (17.19%)	2/59 (3.39%)	4/57 (7.12%)	2/56 (3.57%)
***P* < 3 weight**^¥^	13/46 (28.26%)	3/38 (7.89%)	2/30 (6.67%)	3/24 (12.5%)
***P* > 97 weight**^¥^	1/46 (2.17%)	1/38 (2.63%)	0/30 (0%)	0/24 (0%)
***P* < 3 height**^¥^	23/45 (51.11%)	16/38 (42.11%)	9/30 (30%)	8/24 (33.33%)
***P* > 97 height**^¥^	0/45 (0%)	0/38 (0%)	0/30 (0%)	0/24 (0%)
**BMI** Median (IQR)^¥^	15.70 (15.09–16.90)	16.19 (14.95–17.72)	16.29 (14.48–17.21)	16.30 (15.31–17.41)
***P* < 3 BMI**^§^	1/21 (4.17%)	1/24 (4.17%)	1/31 (3.23%)	1/36 (2.78%)
***P* > 85 BMI**^§^	2/21 (8.33%)	4/24 (16.67%)	4/31 (12.90%)	3/36 (8.33%)
**Pathological psychomotor development**	12/69 (17.39%)	10/65 (15.38%)	9/64 (14.06%)	5/61 (8.20%)
**Correct vaccination**	36/53 (67.92%)	33/47 (70.21%)	34/44 (77.27%)	33/41 (80.49%)
**COVID-19 vaccine**	1/36 (2.78%)	0/30 (0%)	3/35 (8.57%)	5/35 (14.29%)
**COVID-19 vaccine type**	Comirnaty (Pfizer-BioNTech)		**2 × **Comirnaty (Pfizer-BioNTech) CoronoVac (Sinovac Biotech)	**3 × **Comirnaty (Pfizer-BioNTech) **2 × **CoronoVac (Sinovac Biotech)

*ART *Antiretroviral treatment,* P *Percentile,* BMI *Body mass index

^¥^Only in children under 5 years

^§^Only in children over 5 years

^a^According to the CDC clinical classification

Three 3 cases of COVID-19 infection were reported, 1 of which was asymptomatic and 2 manifested only with fever. None required hospital admission or ventilatory support, and the patients received only symptomatic treatment. No cases compatible with pediatric inflammatory multisystemic syndrome related to SARS-CoV-2 (PIMS) were reported.

**Fig. 3:**
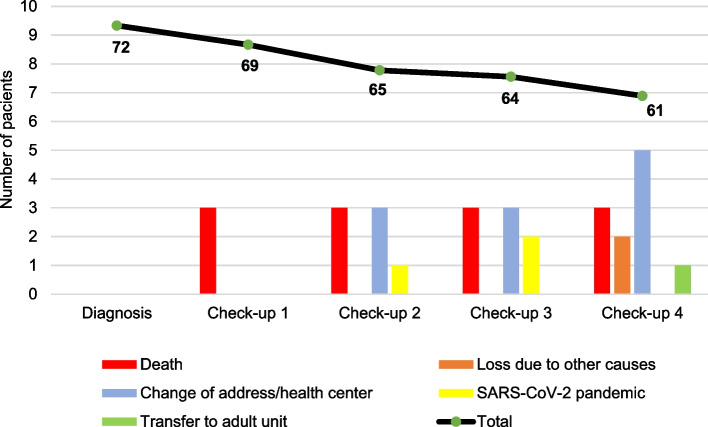
Total number of patients available at each check-up and cause of loss to follow-up *Legend:* *Check-up 1: diagnosis - 6 m* *Check-up 2: 7 m - 12 m* *Check-up 3: 13 m - 24 m* *Check-up 4: 25 m - 36 m*

Three patients died during follow-up. The first died 6 months after diagnosis from opportunistic infections (septic shock and disseminated histoplasmosis). The second patient died from disseminated aspergillosis in the context of advanced HIV disease owing to inadequate follow-up after diagnosis. The third patient died a few days after diagnosis from advanced HIV infection, opportunistic *P. jirovecii* infection, and refractory pneumothorax. One month after the last check-up, a fourth patient also died from acute fulminant hepatitis of unknown origin. Figure 3 details the losses to follow-up and their causes.

Table 2 compares the clinical and analytical characteristics of the children diagnosed with HIV infection early with those in whom the diagnosis was made late.

**Table 2:** Comparison of clinical and analytical characteristics of children with early versus late diagnosis

	**Age < 1 year (*n* = 23)**	**Age > 1 year (*n* = 49)**	***p***
**Opportunistic infections during follow-up**	8/20 (40%)	17/44 (38.64%)	0.9
**Clinical stage N/A during follow-up**	5/19 (26.32%)	16/38 (42.11%)	0.38
**Clinical stage C during follow-up**	12/19 (63.16%)	24/45 (53.33%)	0.47
**Immunological stage 3 throughout follow-up**	6/17 (35.29%)	9/32 (28.13%)	0.6
**Pathological psychomotor development during follow-up**	6/20 (30%)	7/40 (17.5%)	0.27
**CD4 cells at check-up 4 (cells/µl)** Median (IQR), *n* = 56	1175 (770)	1050 (477)	0.08
**CD4 percentage at check-up 4** Median (IQR), *n* = 36	35.7% (9.70%)	33% (10.25%)	0.4
**CD4/CD8 ratio at check-up 4** Median (IQR), *n* = 26	1.14 (0.84)	0.84 (0.56)	0.5
**Viral load at check-up 4 (copies/ml)** Median (IQR), *n* = 59	40 (186)	20 (45)	0.09
**Good adherence at all check-ups**	10/20 (50%)	32/41 (78.04%)	**0.03**
**Toxicity during follow-up**	1/20 (5%)	8/41 (19.51%)	0.13
**Psychosocial care during follow-up**	20/22 (90.91%)	44/49 (89.80%)	1
**Hospitalization during follow-up**	8/21 (38.10%)	9/40 (22.50%)	0.2
**Deaths during follow-up**	0/20 (0%)	3/43 (6.98%)	0.55

*IQR* Interquartile range

Table 3 compares the immunological values (viral load and CD4 cells) between these 2 groups of patients, between the different check-ups, and between the end of follow-up and the beginning of follow-up. In general, no statistically significant differences were observed when comparing the intermediate check-ups, but statistically significant differences were observed when comparing the first with the last check-up (after several years of treatment).

**Table 3:** Evolution of immunovirological values ​​in children with early versus late diagnosis during follow-up

	**Check-up 1** (diagnosis – 6 m)	**Check-up 2** (7 m – 12 m)	***p***	**Check-up 3** (13 m – 24 m)	p*	**Check-up 4** (25 m – 36 m)	p**	p***
**Viral load**	*n* = 68	*n* = 59		*n* = 61		*n* = 59		
**≤ 1 year** (*n* = 23) Median (IQR)	2106 (30212.5)	292 (22800)	0.24	116 (48995)	0.8	40 (240)	0.25	**< 0.01**
**> 1 year (***n* = 49) Median (IQR)	82 (2605)	40 (58)	0.02	40 (46)	0.83	30 (40)	0.78	**< 0.01**
**Global (***n* = 72) Median (IQR)	185 (5972)	40 (263)	0.02	40 (161.5)	0.8	40 (60)	0.4	**< 0.01**
**CD4**	*n* = 65	*n* = 59		*n* = 57		*n* = 56		
**≤ 1 year** (*n* = 23) Median (IQR)	1340 (946)	1517 (1393)	0.3	1082 (795)	0.2	1175 (770)	0.59	0.29
**> 1 year (***n* = 49) Median (IQR)	648 (742.5)	954 (480.75)	< 0.01	957 (563)	0.75	1050 (447)	0.3	**< 0.01**
**Global (***n* = 72) Median (IQR)	875 (808)	1084 (759)	< 0.01	985 (577.5)	0.6	1102 (557.25)	0.7	0.07

*IQR* Interquartile range

*p: check-up 1 vs check-up 2*

p*: check-up 2 vs check-up 3

p**: check-up 3 vs check-up 4

p***: check-up 1 vs check-up 4

In terms of adherence, Table 4 compares the clinical and analytical characteristics between children with poor and adequate adherence.

**Table 4:** Comparison of clinical and analytical characteristics between children with poor (< 90%) and adequate adherence (> 90%)

	**Adherence > 90%** **(*n* = 42)**	**Adherence < 90%** **(*n* = 19)**	***p***
**Opportunistic infections during follow-up**	10/41 (24.4%)	10/18 (55.6%)	**0.02**
**Clinical stage N/A during follow-up**	20/39 (51.3%)	0/16 (0%)	**< 0.01**
**Clinical stage C during follow-up**	15/40 (37.5%)	15/17 (88.2%)	**< 0.01**
**Immunological stage 3 throughout follow-up**	5/34 (14.7%)	8/12 (66.7%)	**< 0.01**
**Pathological psychomotor development during follow-up**	7/42 (16.7%)	5/17 (29.4%)	0.27
**CD4 cells at check-up 4 (cells/µl)** Median (IQR), *n* = 54	1086 (511)	1175 (758)	0.85
**CD4 percentage at check-up 4** Median (IQR), *n* = 25	34.7% (10.6%)	32.5% (16.1%)	0.42
**CD4/CD8 ratio at check-up 4** Median (IQR), *n* = 25	0.97 (0.49)	0.69 (1.04)	0.7
**Viral load at check-up 4 (copies/ml)** Median (IQR), *n* = 57	40 (40)	40 (2395)	0.06
**Viral load < 50 copies/ml at check-up 4**	8/39 (20.5%)	7/18 (38.9%)	0.14
**Toxicity during follow-up**	7/42 (16.7%)	1/17 (5.9%)	0.27
**Psychosocial care during follow-up**	37/42 (88.1%)	17/19 (89.5%)	0.88
**Hospitalization during follow-up**	10/42 (23.8%)	6/17 (35.5%)	0.37
**Deaths during follow-up**	1/42 (2.4%)	0/17 (0%)	0.71

*IQR *Interquartile range

## Discussion

It is estimated that in 2018 in Latin America 36,000 children (0–14 years) were living with HIV and the number of new infections in pediatric age was 4100 [7]. However, few studies have analyzed the frequency of new diagnoses of perinatally transmitted HIV infection in Latin American countries. This study describes the clinical and immunological characteristics, antiretroviral treatment and vaccination of a cohort of 72 patients diagnosed with vertically transmitted HIV infection in 2018 over 3 years of follow-up in 8 Latin American countries (PLANTAIDS).

The natural history of HIV infection has changed dramatically with the advent of ART: infant mortality has fallen, and disease progression has slowed considerably [8]. However, given the aggressiveness of HIV infection in children who do not receive ART and its accelerated progression during the first years of life, the delay in diagnosis and in the initiation of ART leads to HIV-associated deaths. Three patients died in our study, possibly due to late diagnosis and subsequent disease progression. These mortality data are slightly lower than those reported in other Latin American cohorts of children living with HIV [9, 10].

In line with current international guidelines [11–14], ART was initiated in practically all children in the cohort after diagnosis, regardless of the CD4 count at the time of diagnosis. Early initiation of ART (first months after diagnosis) has been shown to reduce mortality, but has also been associated with better immunological recovery [15–17], improved anthropometric parameters [18–20], improved neurocognitive profile [21, 22], reduced viral reservoir [23–25], and long-term reduction in the frequency of non-AIDS events [26]. In our study, we found no statistically significant differences in clinical parameters (clinical/immunological stage, opportunistic infections, hospitalizations, and mortality), immunological parameters (CD4 count, CD4%, CD4/CD8 ratio, and viral load), or psychomotor development between patients diagnosed in the first year of life (early initiation of ART) and those diagnosed later. These differences could be explained by the fact that, in many of the cases we studied, ART was initiated in the advanced stage of the disease, regardless of the age of the child. This highlights the rapid progression of infection in the pediatric age group. On the other hand, we did observe a statistically significant difference between the 2 groups of patients in terms of reduction in viral load between the beginning and the end of follow-up (after 3 years on ART) and increase in CD4 count in those older than 1 year. No statistically significant differences were found in CD4 counts between the first and fourth check-ups in children under 1 year of age, possibly because the range of lymphocytes considered normal decreases with age [27]. 

Based on current international guidelines [11–14], initial ART in children consists of a combination of at least 3 antiretroviral drugs. In accordance with the WHO recommendations [11] in force at the time of the study, we observed less frequent use of AZT (zidovudine) and LPV/r during follow-up, with a progressive trend toward incorporation of new drugs, such as integrase inhibitors (dolutegravir or bictegravir), especially in older patients. However, at the time of this study, some of these "new drugs" were not yet universally available, thus accounting for the use of outdated regimens (such as AZT or raltegravir in patients older than 1 month, EFV in children under 3 years of age…). Moreover, administration of ART to children is hampered by several obstacles [28, 29] that have a direct impact on adherence, as follows: a small number of approved drugs; specific dosages based on age, weight, or pubertal development; the need for special presentations (syrups, dispersible tablets); and the need for adequate palatability. In our study, only 2 patients were able to benefit from the convenience of co-formulated drugs (2 adolescent patients who were prescribed Biktarvy® [FTC-TAF-BIC]). Consistent with recent publications [30], the most frequent adverse effect in our study was lipid profile abnormalities, which was associated with the use of boosted protease inhibitors or EFV.

Pediatric patients living with HIV have an increased risk of more illnesses and more severe illnesses than uninfected children; therefore, appropriate immunization during childhood is important [31]. In general, children should be immunized according to the vaccination schedule in force in each country. Although most vaccines have been shown to be safe and effective in HIV-infected patients [32–34], it should be borne in mind that in the case of vaccines containing live attenuated viruses, the patient must remain asymptomatic and not immunosuppressed so that the vaccine can be administered safely. In our study, a not insignificant proportion of patients were immunized with live attenuated vaccines (12 children received yellow fever vaccine, 10 received varicella vaccine, 40 received at least 1 dose of MMR vaccine, and 23 patients received at least 1 oral dose of polio vaccine), although the immunological status of the patients at the time of administration of these vaccines is unknown. Vaccination against tuberculosis (BCG) is not usually recommended in children with HIV infection owing to the risk of disseminated mycobacterial disease after administration [35]. However, since Latin America is considered a high incidence area for tuberculosis, the BCG vaccine is included in most of the systematic vaccination schedules. In our study, this vaccine was administered to more than a third of the patients. This is not surprising, considering that, at the time of administration of the BCG vaccine (at birth), the diagnosis of HIV infection was unknown in most of the children. Nevertheless, no significant adverse effects were observed in any patient after administration of live attenuated vaccines or BCG vaccine, possibly because the most frequent immunological stage at all check-ups was 1 (not evidence of suppression). These vaccination rates, together with those for the other most common preventable diseases (92.9% against HBV, 87.8% against dTP, 92.7% against Hib, and 90.2% against pneumococcus), are similar to those published in children living with HIV in Latin America [36]. Only 8 patients were vaccinated against SARS-CoV-2 infection.

In children, HIV infection leads to malnutrition, which in turn can lead to growth retardation, although findings differ widely depending on the geographical area and are more accentuated in middle- and low-income countries [37–42]. In our work, 28.3% and 51.1% of children under 5 years of age had, respectively, a weight and height percentile of less than 3 at the first check-up after diagnosis, with a slight improvement in subsequent check-ups, after several months or years in treatment. As for children older than 5 years, the figures are not so striking, and only 4.2% of the patients had a BMI in a percentile lower than 3. Although the delay in growth and lower scores than expected in terms of weight and height are the most frequent observations, some studies also present data on overweight and obesity in children living with HIV [30, 43]. In our case, the highest number of patients under 5 years of age with a weight percentile greater than 97 (2.6%) was obtained at the second check-up, with lower figures found at the other 3 check-ups. At the second check-up, 16.7% of children older than 5 years had a BMI above the 85th percentile, which the WHO considers overweight. Therefore, in our study, we found higher rates of delayed growth (weight and height) in children under 5 years of age (with improvement after starting ART) and a greater tendency to be overweight in children over 5 years of age. This trend toward obesity has also been observed in other studies of children and adults living with HIV in Latin America [44], and could be attributed to the adoption of diets rich in carbohydrates and refined fats. This type of low diversity dietary pattern has been associated with poverty and extreme poverty [45].

The figures observed for opportunistic infections are similar to those presented in other studies [46, 47], with tuberculosis being the most frequent. Confirming a diagnosis of tuberculosis in children is complicated owing to the difficulty in obtaining adequate samples (invasive procedures are usually required). In addition, microbiological tests based on culture or nucleic acid detection have low sensitivity because of the paucibacillary nature of the disease [48]. Thus, in our study, microbiological confirmation was only achieved in 1 patient. In the remaining cases, empirical treatment was established based on the clinical and/or epidemiological context.

Breastfeeding is not generally recommended in children living with HIV owing to the risk of disease transmission [49–51]. According to the WHO, exclusive breastfeeding should be recommended in countries where this strategy is feasible, safe, acceptable, affordable, and sustainable in order to prevent MTCT of HIV. However, in our work, we observed that somewhat less than half of the children were breastfed, and, as discussed above, the diagnosis of HIV infection was unknown in most cases at birth. Consequently, we do not know whether the infection could have been acquired through breastfeeding in these cases, thus emphasizing the importance of HIV screening in pregnancy and childbirth, as well as in cases where the mother's partner has HIV infection.

It is essential to guarantee adequate therapeutic adherence, as this enables viral suppression to be maintained [52]. However, this is not always easy in children. In our study, adherence was appropriate in only 85% of patients at the start of follow-up in our study, although we found statistically significant differences in the number of patients with a more advanced clinical stage and in the reduction in the number of opportunistic infections between children with adequate adherence and those without it.

We recorded only 3 cases of acute SARS-CoV-2 infection, all of which involved asymptomatic or mild symptoms. However, owing to the pandemic, 3 patients were lost to follow-up. One withdrew from clinical follow-up and discontinued ART, leading eventually to disease progression and virological and immunological failure.

## Conclusions

It is important to diagnose HIV infection early and ensure that infected children are monitored appropriately, since early initiation of ART is associated with a decrease in mortality and has multiple clinical and antibody-related benefits, in both the short and the long term.

Even so, despite initiation of ART, patients with advanced disease can die.

It is important to closely monitor weight and nutritional development (insisting on an appropriate diet to prevent nutritional deficits, overweight, and obesity).

In addition, children living with HIV should be properly immunized by improving vaccination coverage and financing the vaccines needed to complete immunization (meningococcal B, meningococcal ACWY, pneumococcal polysaccharide), which are not included in routine immunization schedules.

Adherence to ART is essential for an adequate treatment response, although it can sometimes prove difficult in children. Resources need to be invested in improving adherence through development of more antiretroviral drugs, facilitating access to already approved drugs (as is the case of integrase inhibitors in many Latin American countries), and pharmacological presentations appropriate to the pediatric age group.

Despite the limitations of a small sample, we found that patients with acute SARS-CoV-2 infection did not differ in terms of clinical manifestations or severity from uninfected children, although, indirectly, the COVID pandemic may have influenced their follow-up.

## Data Availability

The datasets used and/or analyzed during the current study are available from the corresponding author on reasonable request.

## Abbreviations

3TC: Lamivudine
ABC: Abacavir
ART: Antiretroviral treatment
AZT: Zidovudine
BCG: Bacillus Calmette-Guérin
BIC: Bictegravir
BMI: Body mass index
CDC: Centers for Disease Control and Prevention
CYTED: Ibero‐American Programme of Science and Technology for Development
dTP: Diphtheria - Pertussis - tetanus
DTG: Dolutegravir
EFV: Efavirenz
FTC: Emtricitabine
HBV: Hepatitis B virus
Hib: *H**a**emophilus* *influenz**ae*
HIV: Human immunodeficiency virus
IGRA: Interferon gamma release assay
IQR: Interquartile range
LF-LAM: Lateral flow urine lipoarabinomannan assay
LPV/r: Lopinavir/ritonavir
MMR: Measles - Mumps - Rubella
MTCT: Mother-to-child transmission
P: Percentile
PLANTAIDS: Paediatric Network for Prevention, Early Detection and Treatment of HIV in Children
REDCap: Research Electronic Data Capture
RTV: Ritonavir
TAF: Tenofovir alafenamide
TDF: Tenofovir disoproxil fumarate
UNAIDS: United Nations Program on HIV/AIDS
VL: Viral load
WHO: World Health Organization
